# Gallbladder Polyp and Cancer Evaluation After Cholecystectomy: A Retrospective Observational Study

**DOI:** 10.7759/cureus.28089

**Published:** 2022-08-17

**Authors:** Ahmet Alyanak, Ferit Aslan

**Affiliations:** 1 Department of General Surgery, Yüksek İhtisas University Medical Park Ankara Hospital, Ankara, TUR; 2 Department of Medical Oncology, Medical Park Ankara Hospital, Ankara, TUR

**Keywords:** polyp surgery, polyp diameter, cholesterol polyp, gallbladder cancer, gallbladder polyp

## Abstract

Introduction

Our aim in this study is to examine the clinical features of gallbladder polyps (GBPs) detected by preoperative ultrasonography (USG) or after cholecystectomy, especially benign and malignancy.

Methods

One thousand seven hundred patients between the ages of 18-80 who were operated on for cholecystectomy between January 1, 2015, and January 1, 2020, in Ankara Batıkent Medicalpark hospital were evaluated in this study. Of these patients, 149 patients who were operated on due to polyp detected in USG, who were accompanied by polyp while being operated on for another reason, or who had a benign or malignant polypoid structure in postoperative pathology were examined. Mann-Whitney U test was used for continuous variables and the chi-square test was used for categorical variables.

Results

The mean age for patients with polyps was 44.6 years (range: 21-78). When the patient distribution was evaluated according to gender, although women were more, they were proportionally close to each other (female 77 (51.7%), male 72 (48.3%)). When the polyps were examined in pathology, 20 (13.4%) patients did not have polyps. Given the other patients, hyperplastic polyps were detected in 15 (10.1%), cholesterol polyps in 104 (69.8%), and adenocarcinomas in five (3.4%) of them. Of the patients with adenocarcinoma, two had T2 and three had T1 tumors. Two of the patients with gallbladder cancer had sessile polyps below 5 mm. Of the malignant cases, four were female and one was male. The median age in malignant cases was 55 years. All malignant cases had single polyps.

Conclusion

In conclusion, in our study, it was observed that most postoperative GBPs were benign - malignancy should come to mind at an advanced age - and was compatible with current malignant polyp risk factors. Contrary to the literature, female gender dominance was present in our malignant cases.

## Introduction

Most gallbladder polyps (GBP) are non-neoplastic and consist of cholesterol or inflammatory polyps on histological examination. However, 1-3% of GBPs are true polyp adenomas with malignant potential [1].

Surgery has been recommended for polyps with a diameter of more than 10 mm since 1990. Other surgical indications are rapid growth and symptomatic polyp [2]. High-risk factors for malignancy include being over 50 years of age, sessile polyp, being of Asian population, and accompanying primary sclerosing cholangitis.

In the European consensus guidelines published in 2017, surgery over 10 mm is recommended if accompanied by the above-mentioned high-risk factors between 6-9 mm [3]. Increased polyp diameter of more than 2 mm during the follow-up period constitutes the indication of cholecystectomy with a moderate level of evidence. The presence of biliary symptoms (right upper quadrant pain, dyspepsia) causes prophylactic cholecystectomy induction regardless of size. The indication of cholecystectomy associated with clinical complaints has been recommended with a low level of evidence [3].

Although gallbladder cancers are rare, they are both poorly prognosed and costly to treat. It is very important that stage 1 gallbladder tumor is detected and curable only with cholecystectomy [4]. For these reasons, performing ultrasound twice a year for GBPs is a cost and benefit-effective issue.

Our aim in this study is to examine the clinical features of GBPs detected by preoperative ultrasonography (USG) or after cholecystectomy, especially benign and malignancy.

## Materials and methods

Patients

One thousand seven hundred patients between the ages of 18-80 who underwent cholecystectomy between January 1, 2015 and January 1, 2020 in our hospital were evaluated in this study. Of these patients, 149 patients who were operated on due to polyp detected in USG, who were accompanied by polyp while being operated on for another reason, or who had a benign or malignant polypoid structure in postoperative pathology were examined. All of these patients underwent a cholecystectomy.

The demographic data of the patients, the polyp diameter associated with the gallbladder on USG, the number of polyps, the presence of hepatosteatosis, and the presence of stones were recorded. In postoperative pathology, polyp pathology and the presence of chronic cholecystitis were recorded.

When the indications of cholecystectomy were examined, the polyp diameter was over 10 mm, it was 6-9 mm and the presence of high-risk factors, symptomatic gallbladder for any reason, presence of stones, and history of acute cholecystitis were detected.

For this study, retrospective study ethical permissions were obtained from the Yüksek İhtisas University Medicalpark Ankara Hospital IRB (Date: 09.08.2022, Approval Number: 3381/2022). The data were anonymized. Care was taken to protect the data of the individuals in this study. This study was conducted in accordance with the Declaration of Helsinki.

Statistical

Statistical analyses were performed using SPSS (Statistical Package for the Social Sciences) version 22 (IBM Corp., Armonk, NY, USA). A p-value of less than 0.05 was considered statistically significant. Mann-Whitney U test was used for continuous variables and the chi-square test was used for categorical variables.

## Results

The number of polyps evaluated after preoperative USG or cholecystectomy in 1700 patients with cholecystectomy in the five-year period was 149 (8.7%). When false positives were left aside in preoperative USG, 129 (7.5%) patients had polyps in the cholecystectomy material. The mean age for patients with polyps was 44.6 years (Range: 21-78). When the patient distribution was evaluated according to gender, although women were more, they were proportionally close to each other (female 77 (51.7%), male 72 (48.3%)) (Table 1).

**Table 1:** General Features

	N (%)
Age (Mean, Range)	44.6 (Range: 21-78)
Gender	
Female	77 (51.7%)
Male	72 (48.3%)
Ultrasound Findings	
Gallbladder Stone	
Yes	64 (43%)
No	85 (57%)
Polyp diameter (Mean mm, Range)	7.79 (Range: 2-52)
Number of polyps	
One	41 (27.5%)
Two	8 (5.4%)
Three or more	50 (33.6%)
No	50 (33.6%)
Hepatosteatosis	
Yes	47 (31.5%)
No	102 (68.5%)
Pathology Findings	
Chronic Cholecystitis	
Yes	46 (30.9%)
No	103 (69.1%)
Polyp	
No polyp	20 (13.4%)
Hyperplastic polyp	15 (10.1%)
Cholesterol polyp	104 (69.8%)
İnflammatory polyp	1 (0.7%)
Low-grade dysplasia	2 (1.3%)
Adenomyoma	1 (0.7%)
Adenocarcinoma	5 (3.4%)
Tubulovillous adenoma	1 (0.7%)

When patients with polyps were examined on ultrasound, the average polyp diameter was 7.79 mm (range: 2-52). Regarding numbers, 41 (27.5%) patients did not have a single polyp, eight (5.4%) had two polyps, 50 (33.6%) had multiple polyps, and the remaining 50 (33.6%) had polyps in ultrasound (postoperative pathology). Of the patients with polyps, 64 (43%) had gallstones, and 47 (31.5%) had hepatosteatosis.

When the polyps were examined in pathology, 20 (13.4%) patients did not have polyps. Considering the others, hyperplastic polyps were detected in 15 (10.1%), cholesterol polyps in 104 (69.8%), and adenocarcinomas in five (3.4%) patients. Of the patients with adenocarcinoma, two had T2 and three had T1 tumors. Two of the patients with gallbladder cancer had sessile polyps below 5 mm. Of the malignant cases, four were female and one was male. In malignant cases, the median age was 55 years (47.51, 55.70, and 78 years, respectively). All malignant cases had single polyps.

In pathologies, 46 (30.9%) patients had chronic cholecystitis. There was no primary sclerosing cholangitis in our patients.

The presence of polyps in postoperative pathology was significantly associated with chronic cholecystitis (< 0.001) and female gender (p: 0.037) in our study (Table 2).

**Table 2:** Chi-square test for categorical variables USG: Ultrasonography

		Pathology polyp		P value
		Yes	No	
USG, polyp	Yes	82	17	0.059
	No	47	3	
USG, gallstones	Yes	55	9	0.842
	No	74	11	
USG, hepatosteatosis	Yes	39	8	0.382
	No	90	12	
Pathology, Chronic cholecystitis	Yes	28	18	<0.001
	No	101	2	
Gender	Female	71	6	0.037
	Male	58	14	

## Discussion

The prevalence of gallbladder polyp ranges from 1.3% to 9.5% in different races and regions. While it has a high prevalence in Asia and Denmark, a lower prevalence has been detected in German society. Studies on gallbladder polyp risk factors have been reported to be excessive in eastern Asian society [5]. Gallbladder polyps are classified as a neoplastic, non-neoplastic polyps or pseudopolyps. Non-neoplastic polyps constitute approximately 70% of gallbladder polyps. Non-neoplastic polyps generally do not require benign and additional treatment [6]. Accordingly, follow-up and treatment of adenomatous polyps with neoplastic potential are very important.

The polyp prevalence in cholecystectomy specimens varies between 2.6% and 12.1%. In 1000 polyp cholecystectomy performed in the USA, 5.4 gallbladder cancer can be prevented [7]. The number of surgeries performed has been pushed to define the correct indication for cholecystectomy due to the possible morbidity, surgical risk, and a low number of cancer cases that can be prevented when the cost is considered. Today, this indication has been defined as polyp over 10 mm, high risk between 6-9 mm (over 50 years of age, presence of primary sclerosing cholangitis, Indian race), fast-growing and symptomatic polyp [3]. In our study, the rate of polyps in cholecystectomy material was 7.5%. Our indications for cholecystectomy were in line with the literature.

Age is a well-defined risk factor for cancer. In a systematic review of age and a prospective study involving 1204 patients, the findings showed that over 50 years of age increased the risk 7-11 times for malignant polyps [8,9]. Malignant polyp development is higher in women than in men. The mean age of malignancy is 58 years [10]. In our study, four of the five malignant cases were in females and the median age of malignancy was 55 years.

Abdominal USG is the best radiological method for detecting gallbladder polyps today. Given that it is cheap and accessible, and its sensitivity and specificity are good is decisive in this regard [5]. In a meta-analysis and systematic literature review conducted on this subject, it was observed that abdominal USG had 84% sensitivity and 96% specificity in these studies involving 15,000 patients [11-12]. In clinical practice, when USG is requested, it should be stated that it is requested for polyp and patients should be directed to experienced radiologists. In the desired abdominal USG for random or other indication, GBP may be overlooked. In our study, no polyp was detected in USG and we can show this as the reason for polyp detection in postoperative pathology.

The frequency of cholesterol polyps constitutes 70-80% of all gallbladder polyps. It is usually in those people less than 50 years old and more common in women. In USG, it is generally smaller than 10 mm, multiple and immobile in position [12-14]. In a meta-analysis evaluating 11,685 patients, the rate of cholesterol polyps was 60.5% pathologically. Of all polyps, 15.2% had adenoma, 11.7% had incidental cancer, 7.1% had adenomyoma, 4.1% had an inflammatory polyp, and 1.4% had hyperplastic polyp [10]. In our study, 15 (10.1%) had hyperplastic polyps, 104 (69.8%) cholesterol polyps, 0.7% inflammatory polyps, 1.3% low-grade neoplasia, 0.7% adenomyoma, 0.7% fog adenomyoma, and 3.4% adenocarcinoma.

The presence of sessile polyps has been associated with gallbladder cancer in various studies. In a study by Yang et al. involving 1976 patients, the findings showed that 71% of sessile polyps were malignant [15,16]. In the study by Bhatt et al., the findings showed that the risk of cancer increased seven times in sessile polyps [8]. Given that the gallbladder wall is thicker than 4 mm was associated with an increased risk of cancer. In the 2017 European consensus guideline, the acceptance of a limit above 10 mm was accepted with a moderate level of evidence. When it is 10 mm higher, the risk of malignancy increases by 25% [3]. In a prospective study involving 689 patients, polyp diameter over 10 mm was an independent risk factor for malignancy [14]. However, it is an important reality that malignancy can also be detected in polyps below 10 mm. In a large series of meta-analyses, malignancy was 8.5% in polyps above 1 cm and 1.2% in polyps below 1 cm. In our study, two (1.5%) patients had sessile polyps over 10 mm, one patient (0.7%) 6-9 mm, and two patients (1.5%) below 5 mm.

In the study conducted by Xu et al., hepatitis B infection was defined as a risk factor for cholecystitis and male gender polyp formation [17]. Sessile morphology has been defined as a risk factor for polyp diameter, gallstones and wet malignant polyps [10,17].

In a meta-analysis conducted on the Asian population, the findings showed that there was no relationship between the fatty liver and polyp. On the contrary, the frequency of polyp increased in patients with high body mass index [10,17]. In our study, although hepatosteatosis was detected in 31.5% of the patients, it did not reach statistical significance. The rate of those with stones in the gallbladder was 43%. Of the five malignant cases, three had stones. Two of our malignant cases were over 70 years of age.

When the limitations of our study are examined, we can say that it is retrospective, it is a single center, and the patients are heterogeneous.

## Conclusions

In conclusion, in our study, it was observed that most postoperative GBPs were benign - malignancy should come to mind at an advanced age - and were compatible with current malignant polyp risk factors. There was a significant difference in favor of the female gender and GBP in patients with chronic cholecystitis. Inconsistent with the studies in the literature, female gender dominance was present in our malignant cases.
